# Verruciform Xanthoma of the Ventral Surface of the Tongue: A Rare Case Report and Literature Review

**DOI:** 10.22038/ijorl.2020.45912.2506

**Published:** 2021-01

**Authors:** Saede Atarbashi-Moghadam, Ali Lotfi, Farnoush Kabiri, Soran Sijanivandi

**Affiliations:** 1 *Department of Oral and Maxillofacial Pathology, School of Dentistry, Shahid Beheshti University of Medical Sciences, Tehran, Iran. *; 2 *Department of Molecular Genetics, Faculty of Biological Sciences, Tarbiat Modares University, Tehran, Iran. *; 3 *Dental Research Center, Research Institute of Dental Sciences, Shahid Beheshti University of Medical Sciences, Tehran, Iran. *

**Keywords:** Benign, Oral cavity, Tongue, Verruciform xanthoma

## Abstract

**Introduction:**

Verruciform xanthoma (VX) is an uncommon benign lesion with the subepithelial accumulation of foamy histiocytes and superficial papillary proliferations with a bright orange hue. This lesion exhibits an oral region predilection. Its clinical differential diagnosis includes verrucous leukoplakia, verrucous carcinoma, squamous papilloma, verruca vulgaris, condyloma accuminatum, squamous cell carcinoma, and fibroepithelial polyp.

**Case Report:**

This report presents a case of VX of the ventral surface of the tongue afflicting a 33-year-old otherwise healthy male.

**Conclusion:**

This case report can be valuable as a consequence of VX rarity and the similarity of its clinical features to papillary lesions. A biopsy is required for its definite diagnosis particularly when it occurs at sites with a high-risk of squamous cell carcinoma development, such as the lateral border and ventral surface of the tongue.

## Introduction

Oral verruciform xanthoma (VX) is a rare benign lesion of unknown pathogenesis with the subepithelial accumulation of lipid-laden histiocytes. Although it is mainly known as an oral disease, some cases of skin and genital lesions have also been reported. The VX is possibly an uncommon reaction or immune response to localized epithelial trauma or damage (1). 

The VX is usually located on the gingiva and manifests itself as a painless, solitary, sharply demarcated, and slightly raised plaque with a papillary, granular, or verrucous surface and a yellowish (2), reddish, or grayish color (1). 

It has a slight male predilection, and the majority of afflicted patients are in their 5^th^ to 7^th^ decades of life (2). The greatest diameter of most reported lesions is smaller than 2 cm (1). 

Microscopically, VX is characterized by papillary epithelial hyperplasia and foamy macrophages in the connective tissue papillae (3). It is usually treated by a conservative surgical excision (1,2). 

As a consequence of VX rarity and similarity of its clinical features to papillary lesions, reporting more of such cases is valuable for the prevention of their overdiagnosis. This report presents a case of VX of the ventral surface of the tongue occurring in a 33-year-old otherwise healthy male. The present case is important due to its rare location, which is one of the most common sites of oral squamous cell carcinoma, and large size.

## Case Report

**Fig 1:**
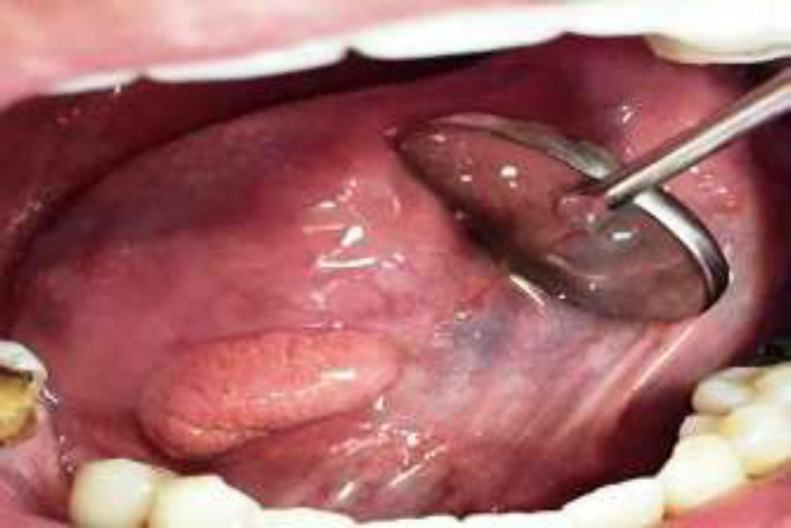
A well-defined oval mass with a tiny papillary surface and a pink to yellowish color in the right ventral aspect of the tongue measuring 2.2×1.2 centimeters

A 33-year-old male patient was referred to an oral pathology center for the evaluation of a well-demarcated, non-tender, pink to yellowish mass with a delicate papillary (roughened) surface on the right ventral surface of the tongue measured at 2.2 × 1.2 cm that had been present at least for 4 years (Fig.1).

The lesion consistency was soft on palpation. There was no evidence of extra-oral lesions or cervical lymph node enlargements, and the laboratory results were normal. With the provisional diagnosis of benign papillary lesions, such as large squamous papilloma, VX, and condyloma acuminatum, an excisional biopsy was performed under local anesthesia. Microscopic sections showed a papillary hyperparakeratotic acanthotic epithelium with parakeratin plugging between the papillary projections. Rete ridges were elongated to a uniform depth. The connective tissue papillae were composed of numerous large macrophages with foamy cytoplasm (xanthoma cells) (^Fig’s 2^,3). The diagnosis of oral VX was confirmed considering clinical and histopathologic features. The patient was free of disease within 15 months following the operation. Written informed consent was obtained from the patient.

**Fig 2:**
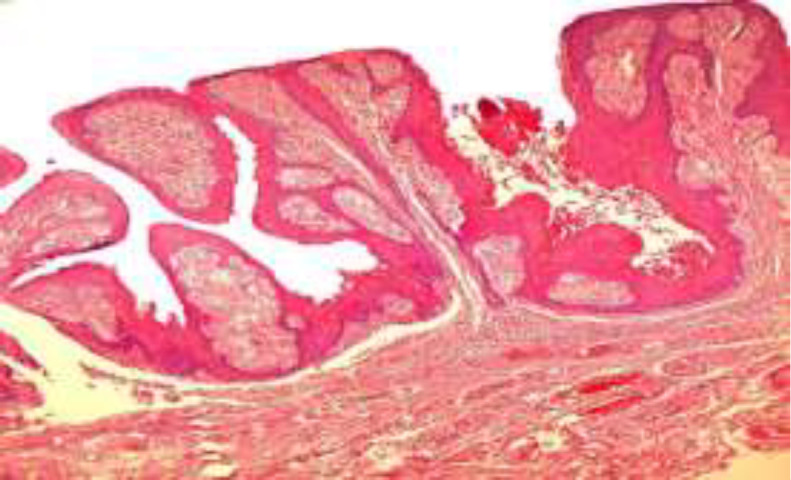
Microscopic sections show hyperparkeratosis with papillary projections and the rete ridges are elongated to a uniform depth. Note the parakeratin plugging between the papillary projections. (H&E ×100).

**Fig 3:**
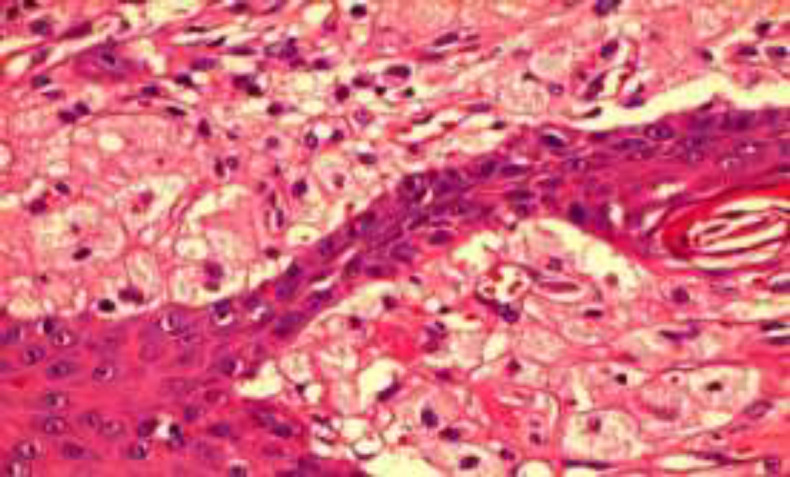
The connective tissue papillae are composed of large histiocytes with foamy cytoplasm (xanthoma cells). (H&E ×400).

## Discussion

The VX is a rare lesion with a frequency of occurrence reported as approximately 0.025% (4). About 75% of all oral VXs arise on the masticatory mucosa of gingiva and hard palate (3). However, the location of the lesion in the current case was the ventral surface of the tongue. The greatest mean dimension of VX in a case series carried out by Yu et al. was reported as 0.8 cm (4); nonetheless, the size in the present case was higher than 2 cm. 

The clinical differential diagnosis of VX includes verrucous leukoplakia, verrucous carcinoma, squamous papilloma, verruca vulgaris, condyloma accuminatum, and squamous cell carcinoma (2-5). Nevertheless, the presence of foamy histiocytes and superficial papillary proliferation with a bright orange hue and neutrophil exocytosis distinguishes it from malignancies (3). Yu et al. (4) reported that oral VXs may show surface ulceration or be similar to a fibroepithelial polyp and only 20% of the cases had a correct initial diagnosis in their study. Furthermore, in a case series conducted by Belknap et al. (6), only 0.53% of oral VXs had a correct clinical diagnosis. This finding highlights the importance of familiarity with the clinical characteristics of VX. Unlike skin xanthoma, no disturbance of lipid metabolism has been reported (2), and the majority of cases do not show hyperlipidemia (5). Blood study in the present case was also within the normal limit. The VX is usually associated with previous epithelial or inflammatory disorders, such as pemphigus vulgaris, lichen planus (LP), and graft versus host disease (GVHD) (3). The present patient also underwent a complete oral examination to rule out LP (Wickham striae). The case also did not mention a history of transplantation, skin disorder, or use of medication. The main theory for VX is that the destruction of epithelial cells results in the breakdown of the phospholipid-rich cell membranes releasing lipids, which are taken up by connective tissue macrophages (3). However, in the present patient, VX occurred in a completely healthy mucosa with no history of trauma or specific mucosal changes in the area. Theofilou et al. (7) explained that VX cases associated with oral LP tend to affect nonkeratinized mucosa and suggested that the inflammation in oral LP may play a pathogenic role in the occurrence of VX. In another study, Getz et al. (8) studied missense mutations in NAD (P)-dependent steroid dehydrogenase-like (NSDHL) gene, which is known to be associated with congenital hemidysplasia with ichthyosiform erythroderma and limb defects syndrome, and showed that they can be recognized in oral VX. Microscopically, VX is characterized by a papillary hyperparakeratotic surface with a bright orange hue; nevertheless, nonpapillary or flat variants have also been reported. The hallmark feature of VX is the existence of large lipid-laden foam cells within the connective tissue papillae which are positive for CD68, macrophage scavenger receptor-I, monocyte chemoattractant protein-I, CCR2, and oxidized low-density lipoprotein (3,9). However, unusual xanthomatous infiltrates may be accompanied by occasional oral polyps and squamous cell carcinomas (10). Disseminated VX with skin and oral and genital mucosal involvement have also been described (11). The treatment of oral VX is a conservative surgical excision (1,2) and recurrence is rare (6). It has a benign course with an excellent prognosis (7). A literature review of tongue VX within 2000 to April 2020 in PubMed database (with adequate data) resulted in the identification of 21 cases in 14 articles, which are presented in Table 1 (2-4,7,8,10-18), including the current case. In the literature review, men and women were equally affected. In a review carried out by Hiraishi et al. (13) on tongue VX, non-Japanese cases showed no sexual predilection; nevertheless, Japanese cases revealed high female tendency. 

In the present review, the mean age of the patients was reported as 45.86 years (range: 13-73 years). Tongue VX mostly occurred in the 6^th^ decade of life, and only two cases were under the age of 20 years. Given that oral cancer is also more common in the elderly (19), the similar age predilection in addition to papillary manifestation makes the differential diagnosis of VX more challenging than oral cancer. The lateral portion of the tongue was the most commonly affected site (50%) followed by the ventral surface (31.81%) and dorsum (18.18%). Moreover, in a large-scale study conducted by Belknap et al. (6), the lateral portion of the tongue was the most common site of tongue VX. 

**Table1:** Published case reports of tongue verruciform xanthoma from 2000 - 2020)

**N**	**Reference**	**Age**	**Sex**	**Site**	**Associated disease**
1	Barrett et al [10],2019	53	m	ventral	-
2	Barrett et al [10],2019	53	f	lateral	lichenoid inflammation
3	Barrett et al [10],2019	54	m	dorsum	Candida infection
4	Getz et al [8] 2019	56	f	lateral	-
5	Biag et al[12], 2019	20	m	lateral	-
6	Tamiolakis et al[2] 2018	56	f	lateral	-
7	Tamiolakis et al[2] 2018	56	m	lateral	-
8	Theofilou et al [7] 2018	56	f	lateral	Oral lichen planus
9	Hiraishi et al[13] 2016	65	f	lateral	-
10	Marques et al[14]2014	73	f	lateral	-
11	Tang et al[11]2014	44	f	ventral	Disseminated VX (skin, oral & genital)
12	Joshi et al[15] 2012	22	m	lateral	-
13	Stoopler et al [16] 2012	68	m	lateral	Oral lichen planus
14	Shahrabi Farahani et al[3] 2011	13	f	dorsum	GVHD
15	Shahrabi Farahani et al[3] 2011	63	m	lateral	GVHD
16	Mete etal[17] 2009	38	f	ventral	-
17	Yu et al [4]2007	42	f	dorsum	-
18	Yu et al[4] 2007	56	m	ventral	-
19	Yu et al[4] 2007	46	m	dorsum	-
20	Yu et al [4]2007	18	f	ventral	-
21	Visintini et al [18]2006	24	m	ventral	-
22	Current case	33	m	ventral	-
					

In the current review, one patient showed disseminated VX with skin and oral and genital involvement (11). There have also been some reports of multiple oral VX (20,21). Among the cases with underlying disease, there were three cases of LP/lichenoid lesion and two cases of GVHD. Theofilou et al. (7) reported that VX cases associated with oral LP tend to affect the tongue and buccal and labial mucosa.

## Conclusion

In conclusion, in the case of an oral papillary or verrucous lesion, VX should be considered in the differential diagnosis. 

A biopsy is required for a definite diagnosis particularly when it occurs at high-risk sites for squamous cell carcinoma development, such as the lateral border or ventral surface of the tongue. Familiarity with these rare lesions leads to the prevention of their overtreatment.

## References

[1] Oliveira PT, Jaeger RG, Cabral LA, Carvalho YR, Costa AL, Jaeger MM (2001). Verruciform xanthoma of the oral mucosa Report of four cases and a review of the literature. Oral Oncol..

[2] Tamiolakis P, Theofilou VI, Tosios KI, Sklavounou-Andrikopoulou A (2018). Oral verruciform xanthoma: Report of 13 new cases and review of the literature. Med Oral Patol Oral Cir Bucal.

[3] Shahrabi Farahani S, Treister NS, Khan Z, Woo SB (2011). Oral verruciform xanthoma associated with chronic graft-versus-host disease: a report of five cases and a review of the literature. Head Neck Pathol.

[4] Yu CH, Tsai TC, Wang JT, Liu BY, Wang YP, Sun A (2007). Oral verruciform xanthoma: a clinicopathologic study of 15 cases. J Formos Med Assoc.

[5] Hu JA, Li Y, Li S (2005). Verruciform xanthoma of the oral cavity: clinicopathological study relating to pathogenesis Report of three cases. APMIS.

[6] Belknap AN, Islam MN, Bhattacharyya I, Cohen DM, Fitzpatrick SG (2020). Oral verruciform xanthoma: A series of 212 cases and review of the literature. Head Neck Pathol.

[7] Theofilou VI, Sklavounou A, Argyris PP, Chrysomali E (22). ral Verruciform Xanthoma within Lichen Planus: A Case Report and Literature Review. Case Rep Dent..

[8] Getz GI, Parag-Sharma K, Reside J, Padilla RJ, Amelio AL (2019). Identification of NSDHL mutations associated with CHILD syndrome in oral verruciform xanthoma. Oral Surg Oral Med Oral Pathol Oral Radiol..

[9] Ide F, Obara K, Yamada H (2008). Cellular basis of verruciform xanthoma: immunohistochemical and ultrastructural characterization. Oral Disease..

[10] Barrett AW, Boyapati RP, Bisase BS, Norris PM, Shelley MJ, Collyer J (2019). Verruciform xanthoma of the oral mucosa: A series of eight typical and three anomalous cases. Int J Surg Pathol..

[11] Tang R, Kopp SA, Cobb C, Halpern AV (2014). Disseminated verruciform xanthoma: a case report. Cutis.

[12] Baig FAH, Luqman M, Vij H, Ibrahim M (2019). Oral verruciform xanthoma of lateral border of tongue- a sheep in wolf’s clothing. J Stomatol Oral Maxillofac Surg..

[13] Hiraishi Y, Tojyo I, Kiga N, Tanimoto K, Fujita S (2016). A case of verruciform xanthoma arising in the tongue. J Clin Diagn Res..

[14] Marques YM, de Andrade CR, Machado de Sousa SC, Navarro CM (2014). Oral verruciform xanthoma: a case report and literature review. Case Rep Pathol.

[15] Joshi R, Ovhal A (2012). Verruciform xanthoma: report of five cases. Indian J Dermatol.

[16] Stoopler ET, Desai B (2012). A tongue mass in a patient with oral lichen planus. J Can Dent Assoc.

[17] Mete O, Kurklu E, Bilgic B, Beka H, Unur M (2009). Flat-type verruciform xanthoma of the tongue and its differential diagnosis. Dermatol Online J.

[18] Visintini E, Rizzardi C, Chiandussi S, Biasotto M, Melato M, Di Lenarda R (2006). Verruciform xanthoma of the oral mucosa Report of a case. Minerva Stomatol.

[19] Akbari ME, Atarbashi Moghadam S, Atarbashi Moghadam F, Bastani Z (2016). Malignant tumors of tongue in Iranian population. Iran J Cancer Prev..

[20] Capocasale G, Panzarella V, Tozzo P, Mauceri R, Rodolico V, Lauritano D, Campisi G (2017). Oral verruciform xanthoma and erythroplakia associated with chronic graft-versus-host disease: a rare case report and review of the literature. BMC Res Notes.

[21] Qi Y, Sun Q, Yang P, Song A (2014). A case of multiple verruciform xanthoma in gingiva. Br J Oral Maxillofac Surg..

